# Chiral Star-Shaped [Co^III^_3_Ln^III^] Clusters with Enantiopure Schiff Bases: Synthesis, Structure, and Magnetism

**DOI:** 10.3390/molecules29143304

**Published:** 2024-07-12

**Authors:** Liudi Ji, Juntao Wang, Zeyu Li, Xiaoming Zhu, Peng Hu

**Affiliations:** 1 School of Nuclear Technology and Chemistry and Biology, Hubei University of Science and Technology, Xianning 437100, China; jiliudi@126.com (L.J.); juntao-wang@hotmail.com (J.W.); lizeyu725@163.com (Z.L.); 2 Hubei Key Laboratory of Radiation Chemistry and Functional Materials, Hubei University of Science and Technology, Xianning 437100, China

**Keywords:** enantiomeric, tetranuclear compound, Schiff base ligand, single-molecule magnet

## Abstract

Two enantiomeric pairs of new 3d–4f heterometallic clusters have been synthesized from two enantiomer Schiff base derivatives: (*R*/*S*)-2-[(2-hydroxy-1-phenylethylimino)methyl] phenol (*R*-/*S*-H_2_L). The formulae of the series clusters are Co_3_Ln(*R*-L)_6_ (Ln = Dy (**1R**), Gd (**2R**)), Co_3_Ln (*S*-L)_6_ (Ln = Dy (**1S**), Gd (**2S**)), whose crystal structures and magnetic properties have been characterized. Structural analysis indicated that the above clusters crystallize in the chiral *P*2_1_3 group space. The central lanthanide ion has a coordination geometry of *D*_3_ surrounded by three [Co^III^(L)_2_]^–^ anions using six aliphatic oxygen atoms of L^2−^ featuring a star-shaped [Co^III^_3_Ln^III^] configuration. Magnetic measurements showed the presence of slow magnetic relaxation with an effective energy barrier of 22.33 K in the Dy^III^ derivatives under a zero-dc field. Furthermore, the circular dichroism (CD) spectra of **1R** and **1S** confirmed their enantiomeric nature.

## 1. Introduction

Chirality plays a significant role in various fields such as biology, chemistry, and materials science. Recent studies have demonstrated that incorporating chiral characteristics into molecular materials represents a desirable strategy for the fabrication of multifunctional molecule-based magnets [1,2,3]. Chiral molecule-based magnets are typically fabricated using enantiopure chiral organic ligands with specific coordination environments, resulting in unique properties such as magnetochiral dichroism (MChD) [4], ferroelectricity [5], magneto-optical Faraday effects [6], and second harmonic generation (SHG) [7]. In terms of molecule-based magnets, Lanthanide (III) ions, especially dysprosium ions, have been widely utilized in the construction SMMs or mononuclear single-ion magnets (SIMs) due to large spin ground states and magnetic anisotropy. Among them, the achievements of SIMs have been particularly fascinating in recent years because they do not need to consider the problem of performance degradation caused by the inconsistent arrangement of the magnetic axes of polymetallic centers [8,9,10], and a series of significant Dy-SIMs with large energy barriers and high magnetic blocking temperatures have also been developed [11,12,13,14]. Most of them, however, are organometallic compounds, which are extremely air-sensitive and should be handled under an inert atmosphere. It is worth noting that the 3d−4f SMMs containing a lanthanide metal ion, together with one or more diamagnetic 3d ions (e.g., Zn^II^, Ca^II^, Al^III^, low-spin Co^III^ or square-planar Ni^II^), can also be regarded as SIMs, and they are often stable and exercisable [15]. 

Chirality is introduced to such systems in order to modulate magnetic anisotropy and also to derive multifunctional properties [16,17]. However, up to now, a limited number of chiral 3d-4f SIM_S_ have been developed—mainly [MLn] or [M_2_Ln] (one or two diamagnetic 3d ion and a lanthanide metal ion) compounds, such as chiral [Zn^II^Ln^III^] [1,18,19,20], [Ni^II^Ln^III^] [19,20], [Co^III^_2_Ln^III^] [6,21], and [Zn^II^_2_Ln^III^] [2,15] constructed by enantiomeric salen or polyol Schiff base ligands. It is possible to adjust the magnetic properties by adjusting the coordination environment of the magnetic center and the magnetic dilution effect by continuously increasing the diamagnetic atom composition, but this faces challenges in design and synthesis because the magnetic center will often increase accordingly during the coordination process [22,23]. At present, as far as we know, there were only two chiral tetranuclear heterometallic [Zn^II^_3_Dy^III^] [24] and [Zn^II^_3_Er^III^] [25] SIMs which emerged with a small energy barrier.

It has been validated that the selection of chelating ligands plays a crucial role in constructing chiral 3d-4f clusters with the desired properties. To meet the different coordination requirements in these complexes, it is widely accepted that chelating ligands should have multiple ligand sites and a flexible backbone [26,27,28,29]. The enantiomeric Schiff base derivatives, (*R*/*S*)-2-[(2-hydroxy-1-phenylethylimino)methyl] phenol (*R*-/*S*-H_2_L), obtained in situ during the reactions of *R*-/*S*-2-phenylglycinol and salicylaldehyde, are suitable for constructing heterometallic clusters due to the distinct abilities of coordination sites. The multiple coordination sites and abilities of the derivatives satisfy the assembly requisitions of SMMs and have been utilized in a series of chiral 3d-based magnetic clusters, such as [Cu^II^_4_] [30], [Fe^III^_4_] [31], and [Co^II^Co^III^_3_] [32]. But very limited 3d–4f or 4f systems have been constructed. After examining the aforementioned 3d clusters structure, we have determined that constructing 3d-4f clusters with six-coordinated 4f ions using this ligand is promising due to the 4f ions’ preference for oxygen atom coordination.

**Scheme 1:**
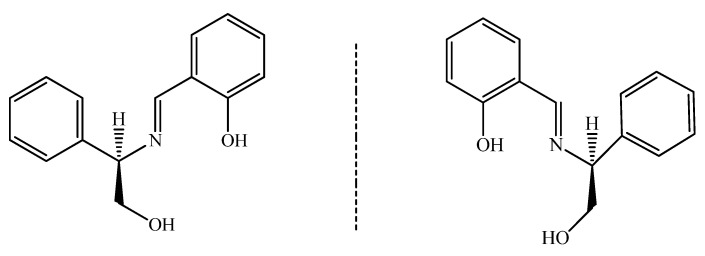
Two enantiomeric Schiff base ligands: *R*-H_2_L and *S*-H_2_L.

Taking into account all of the aforementioned considerations, we have chosen the chiral ligands *R*-/*S*-H_2_L (Scheme 1) to serve as the chelating ligands for investigating the novel 3d–4f magnetic clusters. Herein, two new pairs of chiral 3d–4f heterometallic complexes have been successfully constructed, namely, Co_3_Ln(*R*-L)_6_ (Ln = Dy (**1R**), Gd (**2R**)) and Co_3_Ln (*S*-L)_6_ (Ln = Dy (**1S**), Gd (**2S**)), which exhibit similar star-shaped structures. Magnetic studies revealed the presence of slow magnetic relaxation with an effective energy barrier of 22.33 K in the Dy^III^ derivatives under a zero-dc field. The enantiomeric nature of **1R** and **1S** was confirmed by their circular dichroism (CD) spectra.

## 2. Results and Discussion

### 2.1. Synthesis of Chiral Heterometallic Clusters

All the chiral [Co^III^_3_Ln^III^] clusters were successfully prepared through a simple one-pot method via the reaction of Ln(NO_3_)_3_·6H_2_O (Ln = Dy, Gd), Co(OAc)_2_·4H_2_O, salicylaldehyde-corresponding 2-phenylglycinol, and triethylamine with a rated molar ratio in methanol. The enantiomeric Schiff base ligands in the final structures are generated in situ. Moreover, the Co^III^ ions in the metal core must be produced by the oxidation of Co^II^ salts in the starting materials, and a stoichiometric excess of Dy^III^ precursor may play an important role in promoting the formation of trivalent cobalt in an aerobic reaction because it occupies a favorable position when competing with ligands for coordination [33,34]. The structures of four chiral clusters were all characterized via the analysis of single-crystal X-ray diffraction, and their crystal data and structural parameters are gathered in Table S1.

### 2.2. Structural Characterization

**Figure 1:**
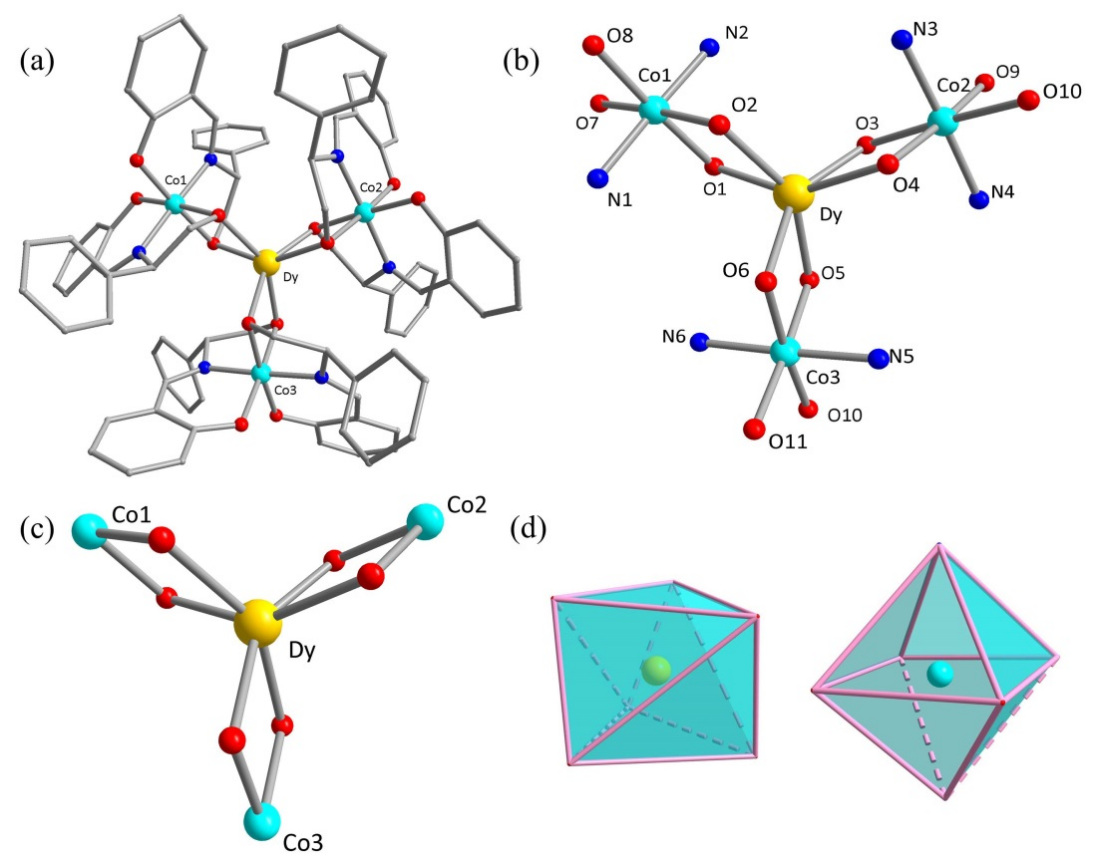
(**a**) Single-crystal X-ray structures of complex **1R**. Hydrogen atoms have been omitted for clarity; (**b**) the [Co^III^_3_Dy^III^] skeleton with coordination environment and atomic labels; (**c**) the star-shaped [Co^III^_3_Dy^III^] core; (**d**) the coordination geometry of Dy^III^ ion (left) and Co^III^ (right) ions in compound **1R**. Color code: Dy^III^ yellow; Co^III^ turquoise; O red; N blue; C gray.

All of the four complexes are isostructural by the analysis of single-crystal and power X-ray diffraction (Table S1, Figure S2), except for the chiral configuration. Herein, only complex **1R** has been structurally described in detail as the example. **1R** crystallizes in the orthorhombic *P*2_1_3 space group, whose asymmetric unit shows the complete structure of star-shaped [Co^III^_3_Dy^III^] in the cluster (Figure 1a). The Dy^III^ ion locates in the center and is surrounded by three [Co^III^(L)_2_]^–^ anions coordinated by six aliphatic oxygen atoms of the ligands L^2–^, while the peripheral three diamagnetic Co^III^ ions are located in the same coordination spheres saturated with two imine-N, two phenoxy-O, and two alcohol hydroxy-O atoms from two ligands L^2–^ (Figure 1b). Each bridging *μ*_2_-O atom originates from the amino alcohol units bind the central Dy^III^ ion and three Co^III^ ions together, forming an attractive [Co^III^_3_Dy^III^] core (Figure 1c). The central Dy^III^ ion and the peripheral three Co^III^ ions are all six-coordinated, showing different coordination configurations. The central Dy^III^ ion adapts the model of twisty tricapped triangular prism, but the Co^III^ ions locate in a slightly distorted octahedral coordination geometry (Figure 1d). Interestingly, The Dy–O bond lengths are 2.179(6) Å for Dy–O (O1, O3, O5) and 2.194(6) Å for Dy–O(O2, O4, O6); they fall within the normal range for bond lengths but are notably smaller than the Dy–O bonds found in similar structures that have been previously reported [35,36,37,38]. The O–Dy–O (O1–Dy–O2, O3–Dy–O4, O5–Dy–O6) angles are 72.1(2)°. The Co–O bond lengths range from 1.901(8) to 1.924(7) A, while the Co–N bond lengths are 1.887(10) and 1.893(9) A. In addition, the distances of adjacent Co···Co and Co···Dy are 5.539(24) Å and 3.198(19) Å. It is predicted that the Dy^III^ ion in the center has a perfect *D*_3_ local symmetry. 

The structure of **1S** is similar to that of **1R**. The coordination geometry of central Dy^III^ ion in **1S** is also a twisty tricapped triangular prism configuration. The Dy–O distances are 2.163(7)Å for Dy–O (O1, O3, O5) and 2.208(6) Å for Dy–O(O2, O4, O6); the O–Dy–O (O1–Dy–O2, O3–Dy–O4, O5–Dy–O6) angles are 71.9(2)°, which are comparable to **1R** with minor differences. The detailed data of bond lengths and angles for **1R** are listed in Table S2.

### 2.3. Magnetic Properties

#### 2.3.1. Static Magnetic Measurements

**Figure 2:**
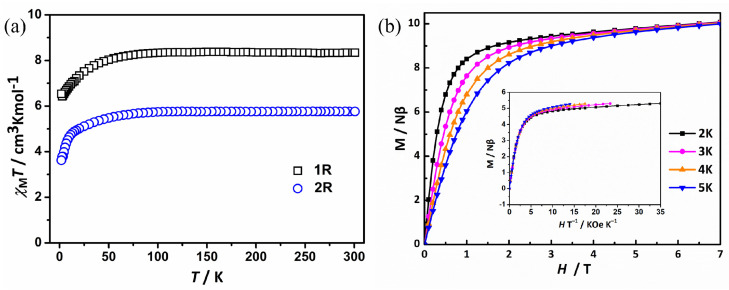
(**a**) Plots of *χ*_M_*T* vs. *T* for **1R** and **2R**; (**b**) plots of *M* vs. *H* and *M* vs. *H*/*T* (inset) for **1R** at 2–5 K. The solid lines are a guide for the eyes.

The magnetic properties of these complexes were investigated by utilizing crystalline samples from the reaction solution. Beforehand, the PXRD tests of the four complexes were carried out to verify their phase purities. The measured and simulated patterns agree well with each other (Figure S2), indicating the purities of these complexes, respectively. The two Gd- or Dy-contained enantiomers exhibited similar magnetic behaviors; and herein, the properties of **1R** and **2R** have been detailed. The variable-temperature magnetic susceptibility measurements have been analyzed in the temperature range of 2−300 K under an applied magnetic field of 1 kOe (Figure 2a). At 300 K, the *χ*_M_*T* values of **1R** and **2R** are 10.34 and 5.79 cm^3^ mol^–1^ K, respectively, significantly less than the expected parameters of 14.17 and 7.88 cm^3^ mol^–1^ K for one uncoupled Ln^III^ ion (Dy^III^, *S* = 5/2, *g* = 4/3; Gd^III^, *S* =7/2, *g* = 2). Upon cooling to 50 K, the *χ*_M_*T* product of **1R** remains constant and then decrease rapidly to 7.97 cm^3^ mol^–1^ K at 2 K. For complex **2R**, the *χ*_M_*T* curve shows a similar tendency. The *χ*_M_*T* decreases smoothly along with the decreasing of the temperature to 25 K; then, it distinctly drops to 3.78 cm^3^ mol^–1^ K at 2 K. These indicate the depopulation of the *M_j_* levels of the Dy(III) ion and, at low temperature, the possibility of weak antiferromagnetic interactions between molecules [15,39]. 

The plots of *M* vs. *H* for **1R** at 2–5 K were obtained (Figure 2b). From 0 to 1.5 T, the values of *M* increase, increasing rapidly, and then slowly attain values above 1.5 T. At 7 T and 2 K, the value of *M* reaches 5.52 *Nβ*, which is much smaller than the theoretical 10 *Nβ*, indicating the unsaturated magnetization. The plot of the *M* vs. *H*/*T* curves (Figure 2b, inset) shows the non-superimposed curves, implying the presence of significant magnetic anisotropy and/or low-lying excited states in these systems, as expected in Dy^III^-based complexes [1,40]. 

#### 2.3.2. Dynamic Magnetic Measurements

**Figure 3:**
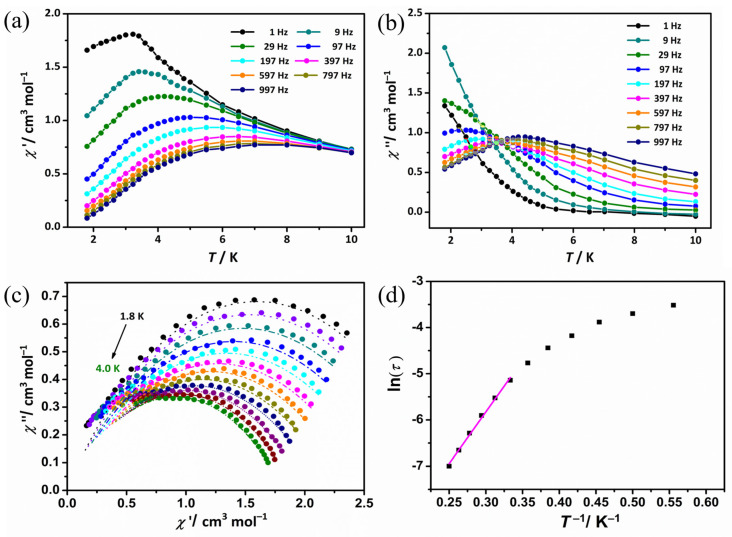
Temperature dependence of the in-phase (**a**) and out-of-phase (**b**) ac susceptibility signals for **1R** under the optimal dc field. The solid lines are a guide for the eyes. (**c**) Cole–Cole plots under the optimal dc field for **1R**. The solid lines indicate the fits using a generalized Debye model. (**d**) The best-fitted curves employing the Arrhenius equation in the high-temperature region.

To probe the magnetic dynamics for complex **1R,** alternating-current (ac) susceptibility measurements were also performed under a zero-dc magnetic field during the temperature range of 1.8–10.0 K with frequencies spanning 1–997 Hz. As shown in Figure S6, a set of temperature-dependent out-of-phase (*χ*″) signals were obtained, indicating slow relaxation of the magnetization of SMM for **1R**, but no well-defined peaks are observed above 1.8 K, which is probably caused by the rapid quantum tunneling of magnetization (QTM), typical of lanthanide magnetic molecules in distorted environments [19]. Therefore, an optimum external field of 2000 Oe was chosen to be used for further magnetic investigations. The SMM behavior of complex **1R** is clearly visible after the application of the external dc field since the presence of resolved peak maxima of both *χ*′ and *χ*″ is observed (Figure 3a,b). 

In order to further verify the magnetodynamics of complex **1R**, a sweep susceptibility test was performed at a temperature range of 1.8 to 4.0 K and a frequency range of 0 to 1000 Hz. As shown in Figure S7, both *χ*′ and *χ*″ of the ac susceptibility of **1R** show obvious frequency-dependent signals with observable peaks, which further indicates that complex **1R** exhibits a typical slow magnetic relaxation behavior of SMM at low temperature.

The results show that QTM has a significant influence on the relaxation process in the low-temperature range but has effect on the slow relaxation process at the high-temperature range, which determines the energy barrier [33]. The Cole–Cole plots for temperatures ranging from 1.8 to 13 K were fitted by employing the generalized Debye model [41] (Figure 3c), and the obtained parameters are given in Table S3. Notably, the values of *α* parameters were in the range of 0.18–0.26, which indicates that there is an almost uniform relaxation process in the measured temperature range. The plots of ln(*τ*) versus *T*^−1^ based on the variable-frequency susceptibility data were first fitted by employing the Arrhenius equation *τ* = *τ*_0_exp(*U*_eff_/*k*_B_*T*) [42] in the high-temperature region (*T*^−1^ < 0.35 K^−1^), obtaining *U*_eff_ = 22.33 K and *τ*_0_ = 3.73 × 10^−6^ s (Figure 3d). The value of *τ*_0_ is within the normal range (10^−5^–10^−12^ s) for the Dy^III^-based SMMs reported previously [43,44].

Summarizing the results of the reported chiral 3d-4f SIMs or analogues (Table 1), it is found that the *U_eff_* value of **1R** is larger than the chiral [Zn_3_Dy] Schiff base SIM with similar structural parameters [24], comparable with the most-reported chiral 3d-4f SIMs [1,2,18,19,20], but much smaller than the chiral [Zn_2_Dy] SIMs [15] with *C*_s_ symmetry of Dy ion and *RRRR*-Dy-*D*_6h_F_12_ (*U*_eff_ = 1833 K) [12]. Meanwhile, it is slightly larger than most achiral [M_3_Dy] SIMs [45,46,47]. These results show that the dominant role in regulating the magnetic properties of 3d-4f SIMs is still that of the axis of anisotropy for the 4f ion, and the role of chirality is limited, but the potential of chirality in inducing multifunctions is fascinating.

### 2.4. Circular Dichroism (CD) Spectrum

**Figure 4:**
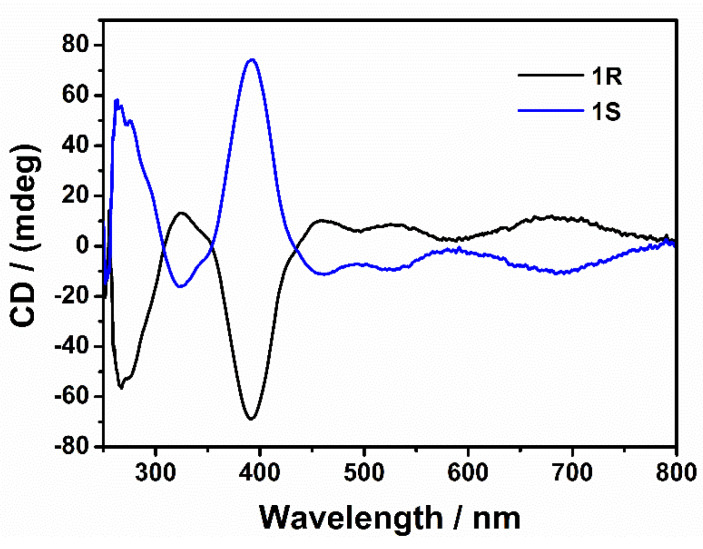
CD spectra of **1R** and **1S** at 298 K (2 × 10^−5^ M, DMF).

The employment of chiral ligands in coordination chemistry often leads to the phenomenon of chirality transfer, which produces structures with pre-determined supramolecular chirality. Enantiomeric *R*-H_2_L/*S*-H_2_L exhibit absorption peaks at 320 nm in DMF solution due to the intramolecular presence of the π-π* and n-π* transitions [31]. In order to study the enantiomeric nature of the above four 3d-4f complexes further, electronic circular dichroism in both solution and a solid state were studied, respectively, with **1R** and **1S** as representatives (Figure 4 and Figure S8). The spectrum of **1R** in DMF solution exhibits a positive Cotton effect at 320 nm and negative Cotton effects at 260 and 390 nm, whereas **1S** shows a mirror image at the same wavelengths. Their solid state circular dichroic chromatography also show a good mirror symmetry relationship, confirming enantiomers with each other.

## 3. Materials and Methods

### 3.1. Reagents and Solutions

Co(OAc)_2_·4H_2_O, Dy(NO_3_)_3_·6H_2_O, Gd(NO_3_)_3_·6H_2_O, triethylamine, salicylaldehyde, (*R*)-(−)-2-phenylglycinol, (*S*)-(+)-2-phenylglycinol, methanol, and petroleum ether were bought from Sinopharm Chemical Reagent (Shanghai, China). All starting materials were employed without further purification.

### 3.2. Instruments

Elemental analyses (C, H and N) were determined on a Perkin-Elmer 2400 elemental analyzer (Waltham, MA, USA). The IR spectra were recorded in range of 400–4000 cm^−1^ on a Nicolet 5DX spectrometer (KBr pellets, Thermo Fisher Scientific, Shanghai, China). Powder X-ray diffraction (PXRD) data were collected over the 2θ range 5–50° using a X‘Pert PRO (Cu Ka, Panalytical Company, Almelo, The Netherlands) automated diffractometer at room temperature, with a step size of 0.02° in the 2θ angle. Magnetic susceptibility measurements were carried out in the temperature range of 2–300 K with a magnetic field of 1000 Oe on a Quantum Design MPMS XL-7 magnetometer (San Diego, CA, USA).

### 3.3. Synthesis of ***1R*** or ***1S***

A mixture of salicylaldehyde (12.2 mg, 0.1 mmol) and (R)-(−)-2-phenylglycinol (13.7 mg, 0.1 mmol)/(S)-(+)-2-phenylglycinol (13.7 mg, 0.1 mmol) was added to a methanolic solution (10 mL) of Co(OAc)_2_·4H_2_O (34.6 mg, 0.20 mmol,) and Dy(NO_3_)_3_·6H_2_O (45.7 mg, 0.1 mmol). After stirring for 0.5 h at room temperature, triethylamine (0.43 mL, 0.2 mmol) was added, and the resulting solution was further stirred for 4 h then filtered, and the filtrate was covered with petroleum ether carefully. After 5 days, dark red block-shaped crystals suitable for single-crystal X-ray diffraction were obtained. The crystalline samples were washed with mother liquor and then washed three times with methanol and dried in a vacuum oven at 60 °C (yield 48% for **1R** and 45% for **1S** based on Dy). What is more, in order to obtain high-quality single crystals, a variety of crystallization methods have been tried, including leaving the filtrate to stand at room temperature or low temperature via slow evaporation without being disturbed, the slow diffusion of ethyl ether, or the addition of n-heptane to the filtrate. All these attempts failed to achieve the desired results.

Elemental anal. Calcd (%) for **1R**: C, 59.69, H, 4.56, N, 4.64; found C, 59.43, H, 4.21, N, 4.88. Elemental anal. Calcd (%) for **1S**: C, 59.69, H, 4.56, N, 4.64; found C, 59.61, H, 4.46, N, 4.75. FTIR (KBr pellet, cm^−1^) for **1R**: 3441(br), 3058(w), 3025(w), 2926(m), 2860(m), 1634(s), 1600(s), 1535(s), 1492(w), 1446(s), 1384(m), 1348(m), 1314(s), 1200(m), 1150(m), 1126(w), 1030(s), 948(w), 898(w), 800(w), 754(s), 704(s), 652(w), 595(w), 537(m), 506(s). FTIR (KBr pellet, cm^−1^) for **1S**: 3440(br), 3058(w), 3025(w), 2926(m), 2860(m), 1634(s), 1600(s), 1535(s), 1492(w), 1445(s), 1384(m), 1348(m), 1314(s), 1201(m), 1150(m), 1126(w), 1029(s), 948(w), 898(w), 800(w), 754(s), 704(s), 653(w), 595(w), 537(m), 505(s).

### 3.4. Synthesis of ***2R*** or ***2S***

The procedure for the synthesis of **2** was similar to **1**, except using Gd(NO_3_)_3_·6H_2_O instead of Dy(NO_3_)_3_·6H_2_O. Dark red block-shaped crystals suitable for single-crystal X-ray diffraction were obtained (yield 42% for **2R** and 30% for **2S** based on Gd). Elemental anal. Calcd (%) for **2R**: C, 59.86, H, 4.58, N, 4.65; found C, 59.63, H, 4.47, N, 4.81 Elemental anal. Calcd (%) for **2S**: C, 59.86, H, 4.58, N, 4.65; found C, 59.55, H, 4.52, N, 4.79. FTIR (KBr pellet, cm^−1^) for **2R**: 3440(br), 3058(w), 3025(w), 2922(m), 2862(m), 1634(s), 1600(s), 1535(s), 1492(w), 1445(s), 1384(w), 1348(m), 1314(s), 1201(m), 1150(m), 1127(w), 1029(s), 948(w), 899(w), 800(w), 754(s), 704(s), 653(w), 596(w), 537(m), 506(s). FTIR (KBr pellet, cm^−1^) for **2S**: 3440(br), 3057(w), 3024(w), 2928(m), 2860(m), 1633(s), 1600(s), 1534(s), 1492(w), 1447(s), 1384(w), 1348(m), 1314(s), 1200(m), 1149(m), 1127(w), 1029(s), 947(w), 898(w), 800(w), 754(s), 704(s), 652(w), 595(w), 537(m), 504(s).

### 3.5. Crystallography

X-ray diffraction data of the four complexes were collected on Bruker diffractometer MD2 of BL17B beamline (MoK*α* radiation source, *λ* = 0.71073 Å) of the National Center for Protein Sciences, Shanghai (NCPSS), at Shanghai Synchrotron Radiation Facility. The HKL data were reduced via the HKL3000 program. The structures of the complexes were solved via direct methods using the SHELXT program [48,49], and the non-hydrogen atoms were located from the trial structure and then refined anisotropically with SHELXTL-2014 using a full-matrix least squares procedure based on *F*^2^ values. The hydrogen atom positions were fixed geometrically at calculated distances and allowed to ride on the parent atoms. CCDC 2348361–2348364 contain the supplementary crystallographic data for this paper. These data can be obtained free of charge from The Cambridge Crystallographic Data Centre.

## 4. Conclusions

In summary, two pairs of chiral star-shaped [Co^III^_3_Ln^III^] complexes derived from enantiomerically pure Schiff bases were obtained via the reaction of salicylaldehyde and 2-phenylglycinol and have been rationally designed and successfully prepared, where the Ln^III^ ions are six-coordinated. Magnetic measurements revealed dominant antiferromagnetic exchange within all the compounds, and due to significant magnetic anisotropy of Dy^III^ ions, the slow relaxation of the magnetization was observed in the Dy^III^ derivatives under a zero-dc field, with the derivatives behaving as zero-field SIMs. The CD spectra confirmed that chiral features had been successfully transferred from the ligand to the entire magnetic molecular system. This work presents a feasible method for constructing multifunctional molecular magnets that contain low-coordinated Ln^III^ ions. These chiral clusters will provide an excellent platform from which to explore synergies among different functions (e.g., magneto-optical Faraday effects, proton conduction, asymmetric catalysis). Further comprehensive theoretical studies on these family SIMs with different coordination symmetries of central ions for magnetostructural correlation remain challenges for the future.

## Figures and Tables

**Table 1:** Structural and magnetic data for the chiral 3d-4f SIMs or analogues.

Complexes	Space Group	Coordination Number of 4f Ion	Symmetry of 4f Ion	*U*_eff_ (K)	*τ*_0_ (s)	Ref.
[Ni^II^Dy^III^ (L ^a^)(NO_3_)_3_]	*P*1	10	−	18.4	7.39 × 10^−6^	[20]
[Ni^II^Dy^III^(L ^b^)(NO_3_)_3_]	*C*2	10	−	9.3	2.1 × 10^−5^	[19]
[Zn^II^Dy^III^(L ^b^)(MeOH)(NO_3_)_3_] ·MeOH	*P*2_1_	10	*C*_2v_	17.7	8.3 × 10^−7^	[19]
[Zn^II^Dy^III^(L ^b^)(H_2_O)(NO_3_)_3_]	*P*2_1_	10	−	11.9/46.1	4.23 × 10^−5^/8.85 × 10^−8^	[1]
[Zn^II^Dy^III^(L ^b^)(OAc)(NO_3_)_2_	*P*2_1_	9	*C*_4v_	19.4/51.8	1.23 × 10^−8^/3.75 × 10^−9^	[18]
[Yb^III^Zn^II^_2_(L ^a^)_2_(H_2_O)_4_](ClO_4_)_3_·5H_2_O	*C*222_1_	8	−	8.94	1.91 × 10^−5^	[2]
[Co^III^_2_Dy^III^(L ^c^)_4_]·Cl_5_·(H_2_O)_2_·(CH_3_O)· CH_3_CH_2_OH	*P*2_1_2_1_2	10	−	−	−	[6]
[Co^III^_2_Dy^III^(L ^d^)_6_(Ac)(H_2_O)]·(ClO_4_)_2_·5H_2_O	*P*2_1_2_1_2	10	−	−	−	[21]
[Dy^III^Zn^II^_2_(L ^e^)Cl_2_(H_2_O)](ClO_4_)·0.5MeOH·0.25H_2_O	*P*1	9	*C*_s_	212.1	7.0 × 10^−10^	[15]
[Dy^III^Zn^II^_2_(L ^e^)Cl_2_ (H_2_O)](CF_3_SO_3_)·0.5MeOH	*P*1	9	*C*_s_	203.5	1.0 × 10^−10^	[15]
[Dy^III^Zn^II^_2_(L ^e^)Br_2_ (H_2_O)](CF_3_SO_3_)·0.25MeOH	*P*1	9	*C*_s_	207.3	1.5 × 10^−10^	[15]
[Er^III^Zn^II^_3_(L ^f^)(OAc)(NO_3_)_2_(H_2_O)_1.5_(MeOH)_0.5_]	*P*2_1_2_1_2_1_	9	*−*	8.1	5.3 × 10^−10^	[25]
[Zn^II^(L ^g^)Cl]_3_Dy^III^·MeOH·0.5H_2_O	*P*1	6	*D*_3_	7.0	−	[24]
[Co^III^_3_Dy^III^ (L ^h^)_6_]	*P*2_1_2_1_2	6	*D*_3_	22.33	3.73 × 10^−6^	This work

^a^ (*R*,*R*)-cyclohexane-1,2-diylbis(azanediyl)-bis(methylene)-bis(2-methoxyphenol)](H_2_L); ^b^ phenol,2,2′-[[(1*R*,2*R*)-1,2-diphenyl-1,2-ethanediyl]bis[(*E*)-nitrilomethylidyne]]bis [6-methoxy])(H_2_L); ^c^ (*R*/*S*)-3-[(2-hydroxybenzylidene)amino]propane-1,2-diol(H_2_L); ^d^ (*R*/*S*)-2-(1-hydroxyethyl)pyridine(H_2_L); ^e^ 2-((E)-((1*R*,2*R*)-2-((E)-2-hydroxy-3-methoxybenzylideneamino)cyclohexylimino)methyl)-6-methoxyphenol(H_2_L); ^f^ (*R*,*R*)-*N*,*N*′-tri(2,3-dihydroxybenzene-1,4-dicarbaldehyde)-1,2-diphenylethylenediamine(H_6_L); ^g^ (2,2’-[1,2-ethanediylbis[(methylimino)methylene]]bis [4,5-dimethylphenol])(H_2_L); ^h^ *R*-2-[(2-hydroxy-1-phenylethylimino)methyl] phenol (H_2_L); ^−^ not mentioned.

## Data Availability

Data will be made available upon reasonable request.

## Supplementary Materials

The following supporting information can be downloaded at https://www.mdpi.com/article/10.3390/molecules29143304/s1: Figure S1: Coordination modes of Ligands *R*-/*S*-H_2_L in this work; Table S1: Crystal data and structure refinements for **1R**, **1S**, **2R**, and **2S**; Table S2 selected bond distances (Å) and angles (°) for **1R**, **1S**, **2R** and **2S**; Figure S2: FT-IR spectra of **1R** (black), **1S** (red), **2R** (blue), and **2S** (green); Figure S3: The simulated X-ray powder diffraction patterns (black) and the experimental ones (red) of compounds **1R**, **1S**, **2R**, and **2S**; Figure S4: Plots of the temperature dependence of *χ*_M_*T* vs. *T* for **1S** and **2S**; Figure S5: Magnetization curve for compound **1S** at different temperatures (2 K, 3 K, 4 K and 5 K); Figure S6: Temperature dependence of the in-phase (χ′) (left) and out-of-phase (χ″) (right) ac susceptibility data for **1R** under a zero-dc field; Figure S7: Frequency dependence of the in-phase (χ′) (left) and out-of-phase (χ″) (right) ac susceptibility data for **1R** under a 2000 Oe applied dc field; Table S3: The parameters obtained by fitting Cole–Cole plot under the optimal dc field for **1R**; Figure S8: The solid-state CD spectra of **1R** and **1S** in KBr pellet at 298 K.
