# Supplementary material for: Chiral Star-Shaped [CoIII3LnIII] Clusters with Enantiopure Schiff Bases: Synthesis, Structure, and Magnetism
Source: Molecules. 2024 Jul 12;29(14):3304. doi: 10.3390/molecules29143304 (PMC11279290; doi:10.3390/molecules29143304)
Supplement: Supplementary file 1 [file molecules-29-03304-s001.zip › Supporting imformation.pdf]

Supporting Information

Chiral Star-Shaped [Co_3Ln] Clusters Based on Enantiomeric Schiff Bases: Synthesis, Crystal Structures, and Magnetic Properties

Liudi Ji, [1,2] Juntao Wang, [1,2] Zeyu Li, [1,2] Xiaoming Zhu, *[1,2] Peng Hu*[1,2]

[1]School of Nuclear Technology and Chemistry & Biology, Hubei University of Science and Technology, Xianning 437100, China

[2]Hubei Key Laboratory of Radiation Chemistry and Functional Materials, Hubei University of Science and Technology, Xianning 437100, China

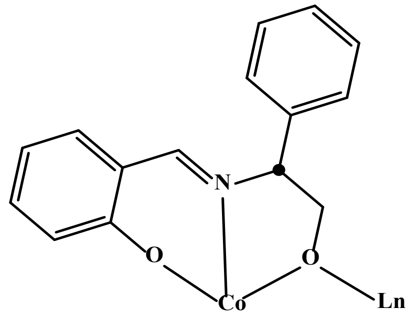

Figure S1. Coordination modes of Ligands *R*-/*S*-H_2L in this work.

Table S1 Crystal data and structure refinements for **1R, 1S, 2R** and **2S.**

Complexes	**1R**	**1S**	**2R**	**2S**
Formula	$C_{90}H_{78}Co_3DyN_6O_{12}$	$C_{90}H_{78}Co_3DyN_6O_{12}$	$C_{90}H_{78}Co_3GdN_6O_{12}$	$C_{90}H_{78}Co_3GdN_6O_{12}$
Formula weight	1774.88	1774.88	1769.63	1769.63
Temp (K)	100.00(10)	100.00(10)	100.01(10)	100.00(10)
Crystal system	cubic	cubic	cubic	cubic
Space group	$P2_13$	$P2_13$	$P2_13$	$P2_13$
a (Å)	21.3246(2)	21.217(3)	21.2867(2)	21.3623(3)
b (Å)	21.3246(2)	21.217(3)	21.2867(2)	21.3623(3)
c (Å)	21.3246(2)	21.217(3)	21.2867(2)	21.3623(3)
α (deg)	90	90	90	90
β (deg)	90	90	90	90
γ (deg)	90	90	90	90
V (Å^3)	9697.1(3)	9551 (3)	9645.5(3)	9645.5(3)
Z	4	4	4	4
D_c (g cm^{-3})	1.214	1.234	1.219	1.206
M (mm^{-1})	8.445	8.574	8.793	8.700
F(000)	3600.0	3612.0	3604.0	3604.0
no. of rflns collected	6587	6487	6537	6613
no. of unique rflns	3565	3513	3539	3579
no. params	331	337	337	337
GOF	1.051	0.993	1.115	0.993
R_1,^{a} wR_2^b [$I > 2\sigma(I)$]	0.0986, 0.2538	0.0896, 0.2403	0.1066, 0.2717	0.0896, 0.2403
R_1, wR_2 (all data)	0.1145, 0.2660	0.1084, 0.2249	0.1198, 0.2824	0.1084, 0.2249
Flack parameter	0.011(7)	0.008(6)	0.013(7)	0.008(6)
CCDC	2348361	2348363	2348362	2348364

$^aR_1 = \sum||F_o| - |F_c||/\sum|F_o|$. $^bwR_2 = [\sum w(F_o^2 - F_c^2)_2/\sum w(F_o^2)_2]^{1/2}$.

Table S2 Selected Bond Distances (Å) and Angles (°) for **1R, 1S, 2R** and **2S**.

Compound	**1R**	**1S**	**1R**	**1S**
Ln-O1	2.179(6)	2.163(7)	2.208(6)	2.167(7)
Ln-O2	2.194(6)	2.208(6)	2.248(6)	2.203(7)
Ln-O3	2.179(6)	2.163(7)	2.208(6)	2.167(7)
Ln-O4	2.194(6)	2.208(6)	2.248(6)	2.203(7)
Ln-O5	2.179(6)	2.163(7)	2.208(6)	2.167(7)
Ln-O6	2.194(6)	2.208(6)	2.248(6)	2.203(7)
O1- Ln-O2	72.1(2)	71.9(2)	71.3(2)	72.1(3)
O1- Ln-O3	91.2(2)	91.8(2)	91.6(2)	91.2(2)
O1- Ln-O4	149.4(3)	149.7(2)	149.4(3)	149.8(3)

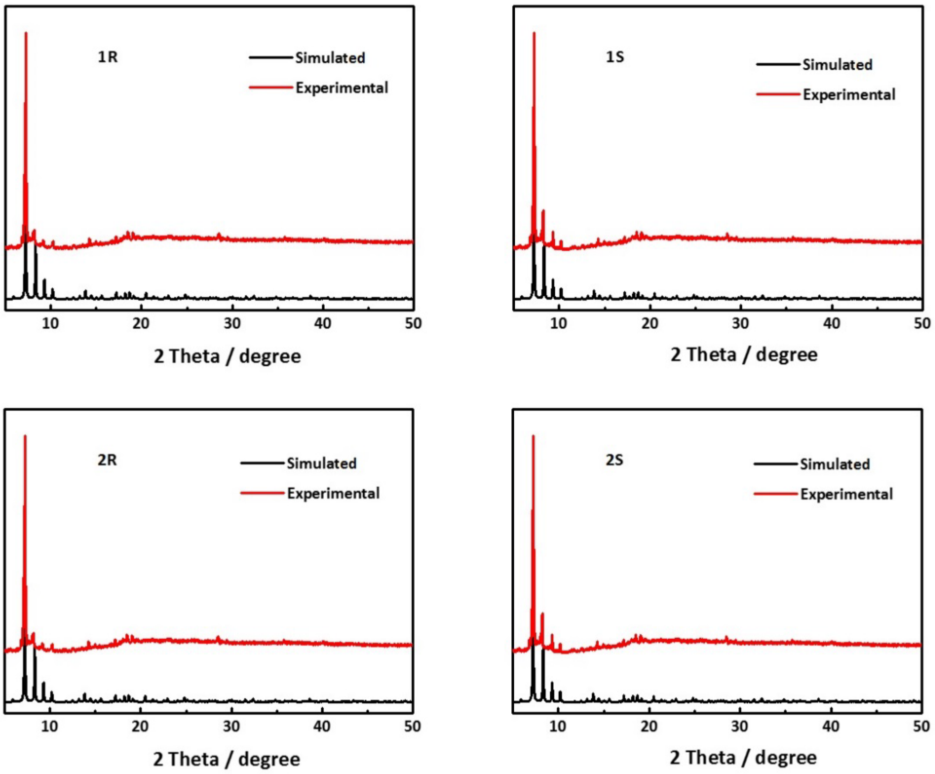

Figure S2. The simulated X-ray powder diffraction patterns (black) and the experimental ones (red) of compounds **1R**, **1S**, **2R** and **2S**. Powder X-ray diffraction (PXRD) of the crystalline samples of complexes **1R**, **1S**, **2R** and **2S** were carried out, and the results are shown in Figure S2. The experimental PXRD patterns are primarily consistent with the the corresponding simulated ones,

highlighting the phase purity of the four complexes. Minor inconsistencies in the intensity and shape of the peaks were observed between the experimental and simulation data due to the different orientations of the crystal samples.

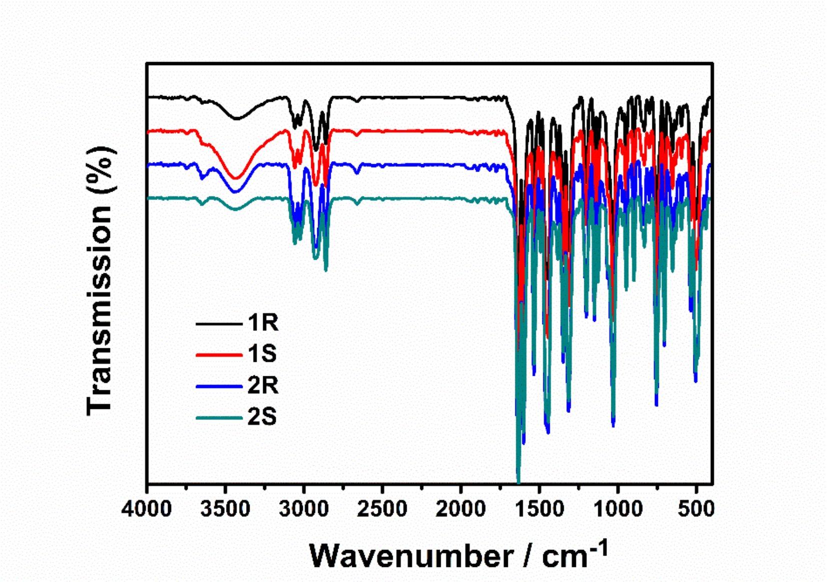

Figure S3. FT-IR spectra of **1R** (black)**, 1S** (red)**, 2R** (blue) and **2S** (green).

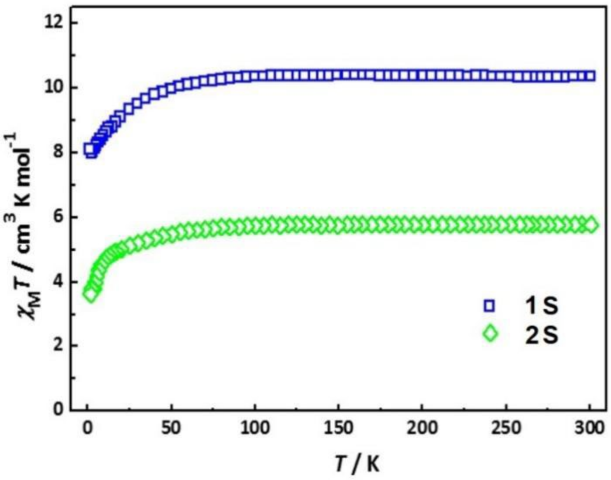

Figure S4. Plots of the temperature dependence of $\chi_M T$ *vs.* T for **1S** and **2S**.

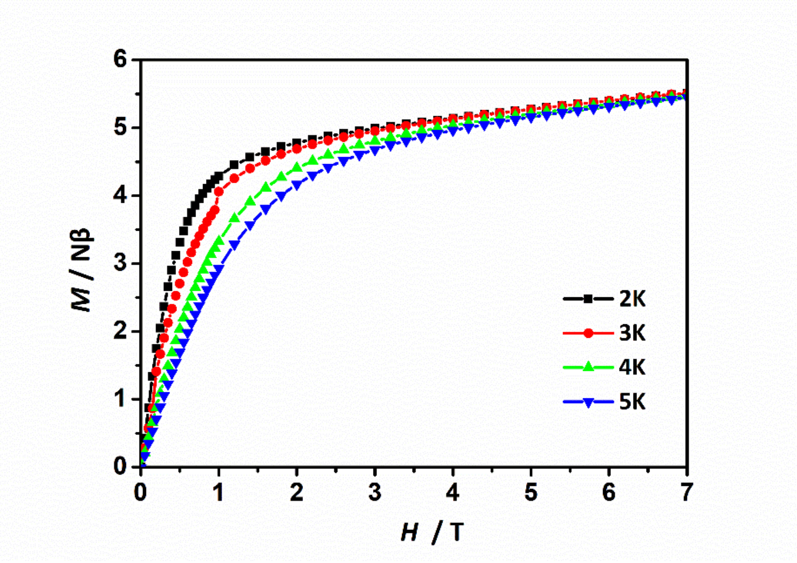

Figure S5. Magnetization curve for compound **1S** at different temperatures (2 K, 3 K, 4 K and 5 K).

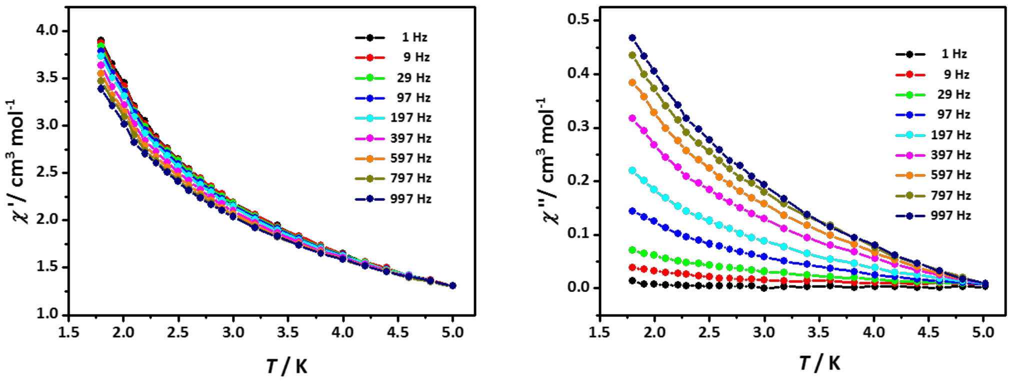

Figure S6. Temperature dependence of the in-phase (χ') (left) and out-of-phase (χ'') (right) ac susceptibility data for **1R** under a zero-dc field.

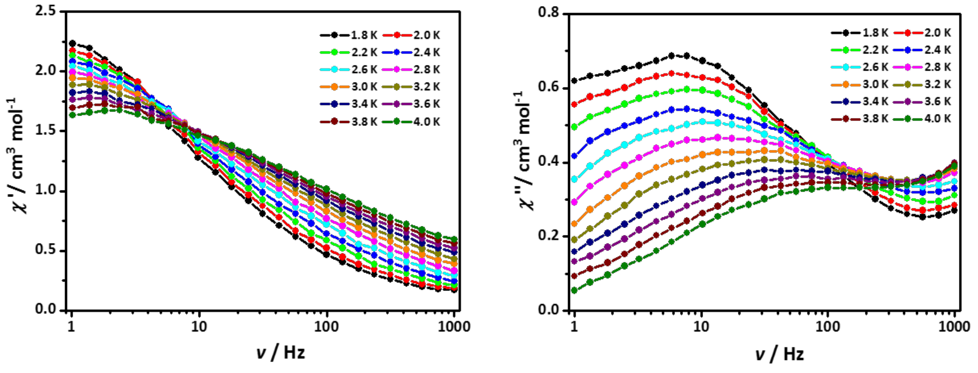

Figure S7. Frequency dependence of the in-phase (χ') (left) and out-of-phase (χ'') (right) ac susceptibility data for **1R** under a 2000 Oe applied dc field.

Table S3. The parameters obtained by fitting Cole-Cole plot under the optimal dc field for **1R**.

T (K)	χ_S	χ_T	τ	α
1.8	0.68	1.76	0.0172	0.26
2.0	0.63	1.68	0.0133	0.25
2.2	0.58	1.52	0.0099	0.23
2.4	0.52	1.35	0.0086	0.22
2.6	0.47	1.21	0.0072	0.22
2.8	0.42	1.13	0.0059	0.23
3.0	0.38	1.06	0.0050	0.21
3.2	0.36	1.02	0.0043	0.20
3.4	0.35	0.96	0.0037	0.19
3.6	0.34	0.90	0.0031	0.20
3.8	0.32	0.85	0.0025	0.19
4.0	0.31	0.82	0.0021	0.18

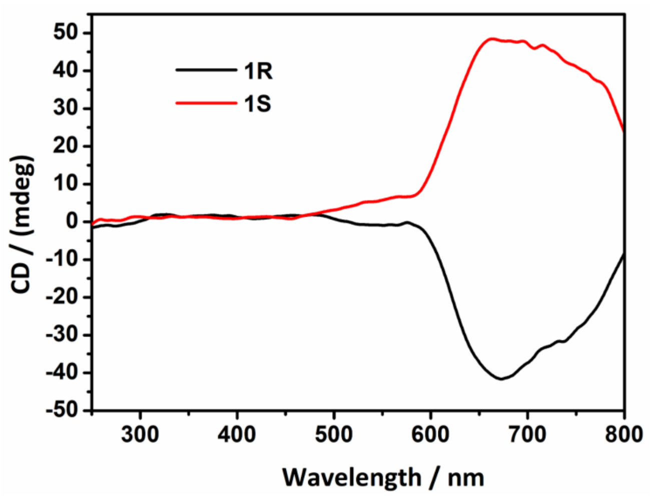

Figure S8. The solid-state CD spectra of **1R** and **1S** in KBr pellet at 298 K.
