# Supplementary material for: Chiral Star-Shaped [CoIII3LnIII] Clusters with Enantiopure Schiff Bases: Synthesis, Structure, and Magnetism
Source: Molecules. 2024 Jul 12;29(14):3304. doi: 10.3390/molecules29143304 (PMC11279290; doi:10.3390/molecules29143304)

checkCIF/PLATON report

You have not supplied any structure factors. As a result the full set of tests cannot be run.

THIS REPORT IS FOR GUIDANCE ONLY. IF USED AS PART OF A REVIEW PROCEDURE FOR PUBLICATION, IT SHOULD NOT REPLACE THE EXPERTISE OF AN EXPERIENCED CRYSTALLOGRAPHIC REFEREE.

No syntax errors found.   CIF dictionary   Interpreting this report

Datablock: p1

```
Bond precision:   C-C = 0.0199 A          Wavelength=1.54184

Cell:              a=21.3246(2)      b=21.3246(2)      c=21.3246(2)
                   alpha=90          beta=90           gamma=90
Temperature:       100 K

                 Calculated                  Reported
Volume           9697.1(3)                   9697.1(3)
Space group      P 21 3                      P 21 3
Hall group       P 2ac 2ab 3                 P 2ac 2ab 3
Moiety formula   C90 H75 Co3 Dy N6 O12       C90 H75 Co3 Dy1 N6 O12
Sum formula      C90 H75 Co3 Dy N6 O12       C90 H75 Co3 Dy N6 O12
Mr               1771.85                     1771.85
Dx,g cm-3        1.214                       1.214
Z                4                           4
Mu (mm-1)        8.445                       8.445
F000             3600.0                      3600.0
F000'            3542.39
h,k,lmax         26,26,26                    26,26,26
Nref             6587[ 3565]                 6587
Tmin,Tmax        0.417,0.509                 0.417,0.509
Tmin'            0.316

Correction method= # Reported T Limits: Tmin=0.417 Tmax=0.509
AbsCorr = MULTI-SCAN

Data completeness= 1.85/1.00       Theta(max)= 73.858

                                                        wR2(reflections)=
R(reflections)= 0.0986( 4592)                           0.2660( 5934)

S = 1.051                   Npar= 331
```

The following ALERTS were generated. Each ALERT has the format
test-name_ALERT_alert-type_alert-level.
Click on the hyperlinks for more details of the test.

Alert level A

```
PLAT602_ALERT_2_A Solvent Accessible VOID(S) in Structure .......          ! Check
```

Author Response: Highly disordered solvent. There is likely other components in the disorder that could not be modeled.

Alert level C

```
CRYSC01_ALERT_1_C The word below has not been recognised as a standard
            identifier.
            crimson
CRYSC01_ALERT_1_C No recognised colour has been given for crystal colour.
DIFMN02_ALERT_2_C  The minimum difference density is < -0.1*ZMAX*0.75
            _refine_diff_density_min given =     -6.077
            Test value =     -4.950
DIFMN03_ALERT_1_C  The minimum difference density is < -0.1*ZMAX*0.75
            The relevant atom site should be identified.
PLAT042_ALERT_1_C Calc. and Reported MoietyFormula Strings  Differ     Please Check
              Calc: C90 H75 Co3 Dy N6 O12
              Rep.: C90 H75 Co3 Dy1 N6 O12
PLAT084_ALERT_3_C High wR2 Value (i.e. > 0.25) ..................       0.27 Report
PLAT098_ALERT_2_C Large Reported Min.  (Negative) Residual Density      -6.08 eA-3
PLAT220_ALERT_2_C NonSolvent   Resd 1  C   Ueq(max)/Ueq(min) Range        3.2 Ratio
PLAT241_ALERT_2_C High   'MainMol' Ueq as Compared to Neighbors of       Dy01 Check
PLAT342_ALERT_3_C Low Bond Precision on  C-C Bonds ...............    0.01993 Ang.
```

Alert level G

```
PLAT003_ALERT_2_G Number of Uiso or Uij Restrained non-H Atoms ...         38 Report
PLAT066_ALERT_1_G Predicted and Reported Tmin&Tmax Range Identical          ? Check
PLAT072_ALERT_2_G SHELXL First  Parameter in WGHT  Unusually Large       0.19 Report
PLAT171_ALERT_4_G The CIF-Embedded .res File Contains EADP Records          1 Report
PLAT177_ALERT_4_G The CIF-Embedded .res File Contains DELU Records          2 Report
PLAT178_ALERT_4_G The CIF-Embedded .res File Contains SIMU Records          6 Report
PLAT186_ALERT_4_G The CIF-Embedded .res File Contains ISOR Records          2 Report
PLAT188_ALERT_3_G A Non-default SIMU Restraint Value has been used     0.0200 Report
PLAT188_ALERT_3_G A Non-default SIMU Restraint Value has been used     0.0100 Report
PLAT188_ALERT_3_G A Non-default SIMU Restraint Value has been used     0.0100 Report
PLAT192_ALERT_3_G A Non-default DELU Restraint Value for SecondPar     0.0200 Report
PLAT232_ALERT_2_G Hirshfeld Test Diff (M-X)  Dy01     --O2       .        7.3 s.u.
PLAT232_ALERT_2_G Hirshfeld Test Diff (M-X)  Dy01     --O3       .        8.0 s.u.
PLAT720_ALERT_4_G Number of Unusual/Non-Standard Labels .........           1 Note
              Dy01
PLAT794_ALERT_5_G Tentative Bond Valency for Dy01      (III)     .       3.64 Info
PLAT794_ALERT_5_G Tentative Bond Valency for Co1       (III)     .       3.24 Info
PLAT860_ALERT_3_G Number of Least-Squares Restraints ............         370 Note
PLAT933_ALERT_2_G Number of HKL-OMIT Records in Embedded .res File         15 Note
               -1  1  5,   3  3  4,  -3  3  4,   2  3  3,   0  0  6,   2  1  3,
```

```
                0  0 14,   2  0  3,   0  1  1,  -4  5  6,   0  2  2,   0  0  4,
                1  1  5,  -2  1  3,   0  5  7,
PLAT941_ALERT_3_G Average HKL Measurement Multiplicity ..........        3.8 Low
```

```
  1 ALERT level A = Most likely a serious problem - resolve or explain
  0 ALERT level B = A potentially serious problem, consider carefully
 10 ALERT level C = Check. Ensure it is not caused by an omission or oversight
 19 ALERT level G = General information/check it is not something unexpected

  5 ALERT type 1 CIF construction/syntax error, inconsistent or missing data
 10 ALERT type 2 Indicator that the structure model may be wrong or deficient
  8 ALERT type 3 Indicator that the structure quality may be low
  5 ALERT type 4 Improvement, methodology, query or suggestion
  2 ALERT type 5 Informative message, check
```

It is advisable to attempt to resolve as many as possible of the alerts in all categories. Often the minor alerts point to easily fixed oversights, errors and omissions in your CIF or refinement strategy, so attention to these fine details can be worthwhile. In order to resolve some of the more serious problems it may be necessary to carry out additional measurements or structure refinements. However, the purpose of your study may justify the reported deviations and the more serious of these should normally be commented upon in the discussion or experimental section of a paper or in the "special_details" fields of the CIF. checkCIF was carefully designed to identify outliers and unusual parameters, but every test has its limitations and alerts that are not important in a particular case may appear. Conversely, the absence of alerts does not guarantee there are no aspects of the results needing attention. It is up to the individual to critically assess their own results and, if necessary, seek expert advice.

Publication of your CIF in IUCr journals

A basic structural check has been run on your CIF. These basic checks will be run on all CIFs submitted for publication in IUCr journals (*Acta Crystallographica*, *Journal of Applied Crystallography*, *Journal of Synchrotron Radiation*); however, if you intend to submit to *Acta Crystallographica Section C* or *E* or *IUCrData*, you should make sure that full publication checks are run on the final version of your CIF prior to submission.

Publication of your CIF in other journals

Please refer to the *Notes for Authors* of the relevant journal for any special instructions relating to CIF submission.

PLATON version of 06/01/2024; check.def file version of 05/01/2024

Datablock p1 - ellipsoid plot

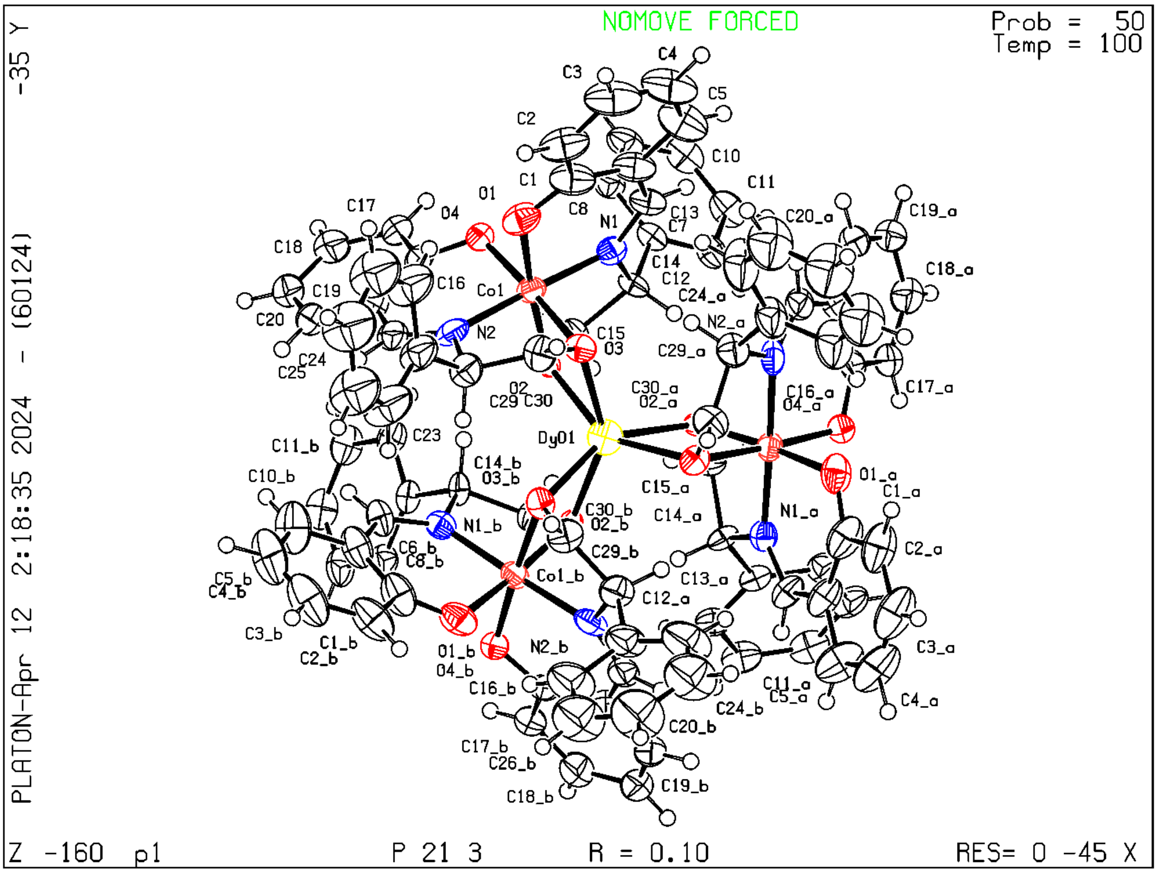

Supplement: Supplementary file 1 [file checkcif-1.pdf › Check cif/checkcif-1.pdf]
