# Supplementary material for: Chiral Star-Shaped [CoIII3LnIII] Clusters with Enantiopure Schiff Bases: Synthesis, Structure, and Magnetism
Source: Molecules. 2024 Jul 12;29(14):3304. doi: 10.3390/molecules29143304 (PMC11279290; doi:10.3390/molecules29143304)

checkCIF/PLATON report

You have not supplied any structure factors. As a result the full set of tests cannot be run.

THIS REPORT IS FOR GUIDANCE ONLY. IF USED AS PART OF A REVIEW PROCEDURE FOR PUBLICATION, IT SHOULD NOT REPLACE THE EXPERTISE OF AN EXPERIENCED CRYSTALLOGRAPHIC REFEREE.

No syntax errors found. CIF dictionary Interpreting this report

Datablock: p3

```
Bond precision:   C-C = 0.0191 A          Wavelength=1.54184

Cell:               a=21.217(2)      b=21.217(2)      c=21.217(2)
                    alpha=90         beta=90          gamma=90
Temperature:        100 K
```

	Calculated	Reported
Volume	9551(3)	9551(3)
Space group	P 21 3	P 21 3
Hall group	P 2ac 2ab 3	P 2ac 2ab 3
Moiety formula	C90 H78 Co3 Dy N6 O12	C90 H78 Co3 Dy N6 O12
Sum formula	C90 H78 Co3 Dy N6 O12	C90 H78 Co3 Dy N6 O12
Mr	1774.88	1774.88
Dx,g cm-3	1.234	1.234
Z	4	4
Mu (mm-1)	8.574	8.574
F000	3612.0	3612.0
F000'	3554.39	
h,k,lmax	26,26,26	26,26,26
Nref	6487[3513]	6487
Tmin,Tmax	0.411,0.504	0.420,0.511
Tmin'	0.311	

```
Correction method= # Reported T Limits: Tmin=0.420 Tmax=0.511
AbsCorr = MULTI-SCAN

Data completeness= 1.85/1.00       Theta(max)= 73.928

R(reflections)= 0.0896( 4499)      wR2(reflections)= 0.2403( 5944)

S = 0.993                  Npar= 337
```

The following ALERTS were generated. Each ALERT has the format
test-name_ALERT_alert-type_alert-level.
Click on the hyperlinks for more details of the test.

Alert level A

```
PLAT602_ALERT_2_A Solvent Accessible VOID(S) in Structure .......          ! Check
```

Author Response: Highly disordered solvent. There is likely other components in the disorder that could not be modeled.

Alert level C

```
CRYSC01_ALERT_1_C The word below has not been recognised as a standard
            identifier.
            crimson
CRYSC01_ALERT_1_C No recognised colour has been given for crystal colour.
PLAT220_ALERT_2_C NonSolvent   Resd 1  C   Ueq(max)/Ueq(min) Range        3.5 Ratio
PLAT234_ALERT_4_C Large Hirshfeld Difference C27      --C28      .       0.16 Ang.
PLAT342_ALERT_3_C Low Bond Precision on  C-C Bonds ..............    0.01907 Ang.
```

Alert level G

```
PLAT003_ALERT_2_G Number of Uiso or Uij Restrained non-H Atoms ...          6 Report
PLAT012_ALERT_1_G N.O.K.   _shelx_res_checksum Found in CIF ......     Please Check
PLAT072_ALERT_2_G SHELXL First  Parameter in WGHT  Unusually Large       0.18 Report
PLAT178_ALERT_4_G The CIF-Embedded .res File Contains SIMU Records          2 Report
PLAT186_ALERT_4_G The CIF-Embedded .res File Contains ISOR Records          3 Report
PLAT188_ALERT_3_G A Non-default SIMU Restraint Value has been used     0.0100 Report
PLAT188_ALERT_3_G A Non-default SIMU Restraint Value has been used     0.0100 Report
PLAT232_ALERT_2_G Hirshfeld Test Diff (M-X)  Dy1      --O2       .        7.0 s.u.
PLAT721_ALERT_1_G Bond    Calc     0.96000, Rep     0.97000 Dev...       0.01 Ang.
              C30      -H30A           1_555   1_555 ........     #   36 Check
PLAT721_ALERT_1_G Bond    Calc     0.96000, Rep     0.97000 Dev...       0.01 Ang.
              C30      -H30B           1_555   1_555 ........     #   37 Check
PLAT721_ALERT_1_G Bond    Calc     0.96000, Rep     0.97000 Dev...       0.01 Ang.
              C15      -H15A           1_555   1_555 ........     #   68 Check
PLAT721_ALERT_1_G Bond    Calc     0.96000, Rep     0.97000 Dev...       0.01 Ang.
              C15      -H15B           1_555   1_555 ........     #   69 Check
PLAT794_ALERT_5_G Tentative Bond Valency for Dy1       (III)     .       3.40 Info
PLAT794_ALERT_5_G Tentative Bond Valency for Co1       (III)     .       3.39 Info
PLAT860_ALERT_3_G Number of Least-Squares Restraints .............         48 Note
PLAT933_ALERT_2_G Number of HKL-OMIT Records in Embedded .res File          5 Note
               -1  2  4,   0  2  2,   1  1  5,   1  0  7,   2  0  3,
PLAT941_ALERT_3_G Average HKL Measurement Multiplicity ..........         4.0 Low
PLAT982_ALERT_1_G The Dy-f'=   -8.8380 Deviates from  IT-value =      -9.8046 Check
PLAT983_ALERT_1_G The Dy-f"=   11.9157 Deviates from  IT-Value =       9.8477 Check
```

1 **ALERT level A** = Most likely a serious problem - resolve or explain
0 **ALERT level B** = A potentially serious problem, consider carefully
5 **ALERT level C** = Check. Ensure it is not caused by an omission or oversight
19 **ALERT level G** = General information/check it is not something unexpected

```
9 ALERT type 1 CIF construction/syntax error, inconsistent or missing data
6 ALERT type 2 Indicator that the structure model may be wrong or deficient
5 ALERT type 3 Indicator that the structure quality may be low
3 ALERT type 4 Improvement, methodology, query or suggestion
2 ALERT type 5 Informative message, check
```

It is advisable to attempt to resolve as many as possible of the alerts in all categories. Often the minor alerts point to easily fixed oversights, errors and omissions in your CIF or refinement strategy, so attention to these fine details can be worthwhile. In order to resolve some of the more serious problems it may be necessary to carry out additional measurements or structure refinements. However, the purpose of your study may justify the reported deviations and the more serious of these should normally be commented upon in the discussion or experimental section of a paper or in the "special_details" fields of the CIF. checkCIF was carefully designed to identify outliers and unusual parameters, but every test has its limitations and alerts that are not important in a particular case may appear. Conversely, the absence of alerts does not guarantee there are no aspects of the results needing attention. It is up to the individual to critically assess their own results and, if necessary, seek expert advice.

Publication of your CIF in IUCr journals

A basic structural check has been run on your CIF. These basic checks will be run on all CIFs submitted for publication in IUCr journals (*Acta Crystallographica*, *Journal of Applied Crystallography*, *Journal of Synchrotron Radiation*); however, if you intend to submit to *Acta Crystallographica Section C* or *E* or *IUCrData*, you should make sure that full publication checks are run on the final version of your CIF prior to submission.

Publication of your CIF in other journals

Please refer to the *Notes for Authors* of the relevant journal for any special instructions relating to CIF submission.

PLATON version of 06/01/2024; check.def file version of 05/01/2024

Datablock p3 - ellipsoid plot

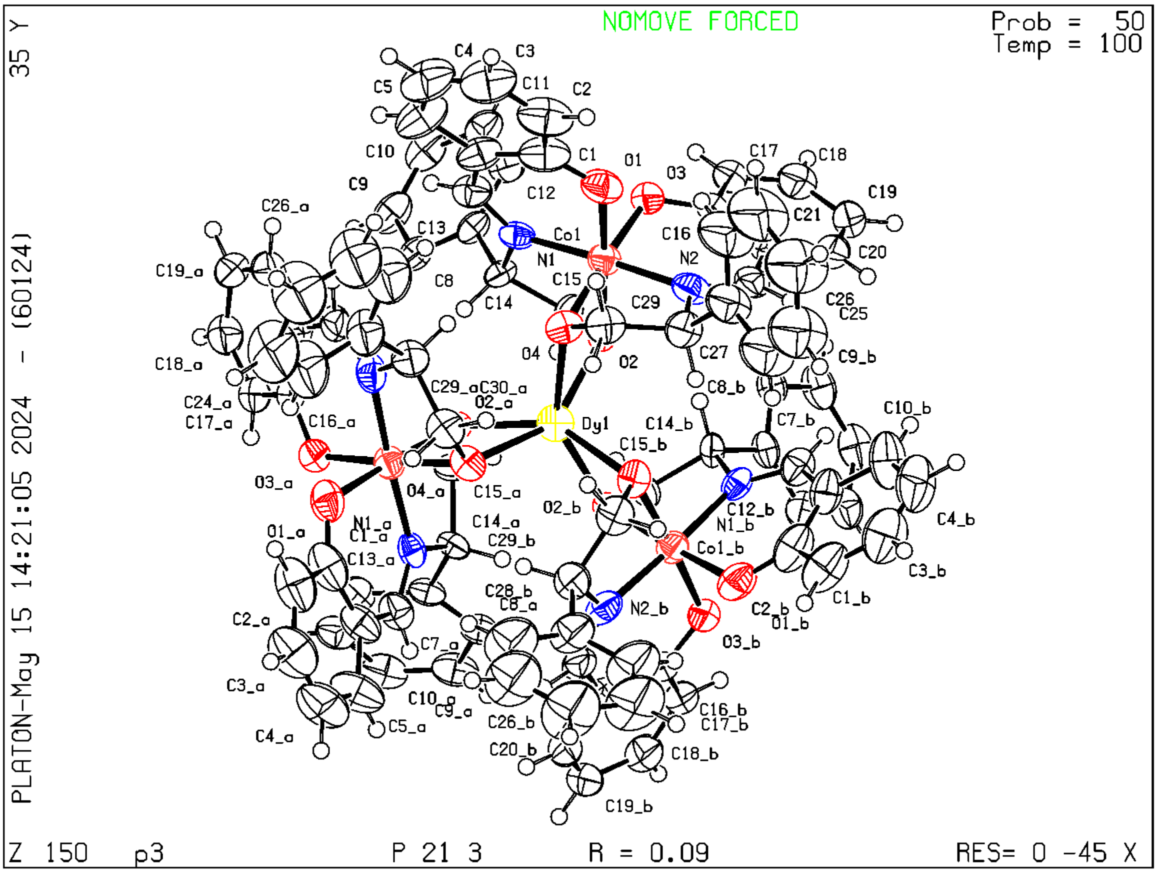

Supplement: Supplementary file 1 [file checkcif-3.pdf › Check cif/checkcif-3.pdf]
